# 5-aminolevulinic acid, fluorescein sodium, and indocyanine green for glioma margin detection: analysis of operating wide-field and confocal microscopy in glioma models of various grades

**DOI:** 10.3389/fonc.2023.1156812

**Published:** 2023-05-23

**Authors:** Evgenii Belykh, Liudmila Bardonova, Irakliy Abramov, Vadim A. Byvaltsev, Talgat Kerymbayev, Kwanha Yu, Debbie R. Healey, Ernesto Luna-Melendez, Benjamin Deneen, Shwetal Mehta, James K. Liu, Mark C. Preul

**Affiliations:** ^1^ The Loyal and Edith Davis Neurosurgical Research Laboratory, Department of Neurosurgery, Barrow Neurological Institute, St. Joseph’s Hospital and Medical Center, Phoenix, AZ, United States; ^2^ Department of Neurosurgery, New Jersey Medical School, Rutgers University, Newark, NJ, United States; ^3^ Department of Neurosurgery, Irkutsk State Medical University, Irkutsk, Russia; ^4^ Department of Neurosurgery, JSC “National Scientific Center of Neurosurgery”, Nur-Sultan, Kazakhstan; ^5^ Center for Cell and Gene Therapy, Baylor College of Medicine, Houston, TX, United States; ^6^ Department of Research Imaging, Barrow Neurological Institute, St. Joseph’s Hospital and Medical Center, Phoenix, AZ, United States; ^7^ Ivy Brain Tumor Research Center, Department of Neurosurgery, Barrow Neurological Institute, St. Joseph’s Hospital and Medical Center, Phoenix, AZ, United States

**Keywords:** 5-aminolevulinic acid, fluorescence guided surgery, fluorescein sodium, glioma, indocyanine green, laser scanning microscopy, protoporphyrin IX

## Abstract

**Introduction:**

Surgical resection remains the first-line treatment for gliomas. Several fluorescent dyes are currently in use to augment intraoperative tumor visualization, but information on their comparative effectiveness is lacking. We performed systematic assessment of fluorescein sodium (FNa), 5-aminolevulinic acid (5-ALA)–induced protoporphyrin IX (PpIX), and indocyanine green (ICG) fluorescence in various glioma models using advanced fluorescence imaging techniques.

**Methods:**

Four glioma models were used: GL261 (high-grade model), GB3 (low-grade model), and an *in utero* electroporation model with and without red fluorescence protein (IUE +RFP and IUE -RFP, respectively) (intermediate-to-low-grade model). Animals underwent 5-ALA, FNa, and ICG injections and craniectomy. Brain tissue samples underwent fluorescent imaging using a wide-field operative microscope and a benchtop confocal microscope and were submitted for histologic analysis.

**Results:**

Our systematic analysis showed that wide-field imaging of highly malignant gliomas is equally efficient with 5-ALA, FNa, and ICG, although FNa is associated with more false-positive staining of the normal brain. In low-grade gliomas, wide-field imaging cannot detect ICG staining, can detect FNa in only 50% of specimens, and is not sensitive enough for PpIX detection. With confocal imaging of low-intermediate grade glioma models, PpIX outperformed FNa.

**Discussion:**

Overall, compared to wide-field imaging, confocal microscopy significantly improved diagnostic accuracy and was better at detecting low concentrations of PpIX and FNa, resulting in improved tumor delineation. Neither PpIX, FNa, nor ICG delineated all tumor boundaries in studied tumor models, which emphasizes the need for novel visualization technologies and molecular probes to guide glioma resection. Simultaneous administration of 5-ALA and FNa with use of cellular-resolution imaging modalities may provide additional information for margin detection and may facilitate maximal glioma resection.

## 1 Introduction

Malignant gliomas are the most common primary brain tumors found in adults. The prognosis is poor even for individuals with early stage cancers, with median patient survival of approximately 1 year (1–4). Current treatment strategies consist of surgical resection with adjuvant radiation and chemotherapy. Studies conducted during the past 20 years have demonstrated that the extent of tumor resection is strongly correlated with average survival time and overall prognosis (1–3, 5–8). In an attempt to optimize the extent of tumor resection, techniques utilizing fluorescent agents have been trialed against standard operative microscope visualization under white light. Fluorescence-guided resection (FGR) has become an active topic of research because of studies showing that, compared with conventional intraoperative neuronavigation, FGR improved the efficacy of tumor identification and was associated with increased rates of gross total resection with the aid of 5-aminolevulinic acid (5-ALA) and fluorescein sodium (FNa) fluorescence, which allowed surgeons to better discriminate marginal areas composed of resectable tumor (1–6, 8–10).

In 1948, Moore et al. documented the earliest fluorescence visualization technique in tumors using intravenous FNa (11). With this technique, they achieved a sensitivity >95% for identifying tumors, noting that brain tumor tissue invariably glowed a brilliant yellow to the naked eye. It was noted subsequently that FNa is not incorporated into tumor cells, but exposes areas of brain tumor–associated disrupted blood-brain barrier (BBB) (7, 8). This characteristic allows for the accumulation of FNa in the extracellular space of tumor tissue, whereas FNa in theory remains virtually undetectable in normal brain tissue. At relatively high doses (20 mg/mL), FNa is readily visible to the naked eye or with a simple surgical microscope (8). With novel filters, FNa fluorescence can be detected at lower doses. A multicenter study demonstrated that FGR of brain tumors with low-dose FNa and a special operating microscope filter allowed gross total resection in 181 (91.4%) of 198 patients (12), with sensitivity and specificity of identifying high-grade glioma tissue of 80.8% and 79.1%, respectively (13).

An increasingly popular FGR technique uses 5-ALA, a nonfluorescent prodrug metabolite of heme synthesis that causes fluorescent protoporphyrin IX (PpIX) to accumulate in cancerous brain tissue (2, 5). This technique requires a special intraoperative optical system with blue excitation light and optical filters for visualization of the red fluorescence. In 1998, Stummer et al. demonstrated 85% sensitivity, 100% specificity, and 90% accuracy in detecting malignant gliomas using 5-ALA (5). In particular, a subsequent study by Stummer et al. showed that the use of 5-ALA not only increased rates of gross total resection, but extended survival rates by up to 6 months (3). 5-ALA was suggested to be better than FNa for detecting tumor cells in boundary zones of glioblastoma (14).

Another fluorophore commonly used in neurosurgery, indocyanine green (ICG), has also been trialed for FGR of brain tumors (15). Potential for intracellular accumulation of ICG has been shown previously in studies of experimental glioblastoma (16, 17). A second window ICG (SWIG) fluorescence technique has been recently described by Lee et al., who reported a sensitivity of 98% and specificity of 45% for identification of glioma tissue (18).

The objective of our study was to perform a detailed systematic assessment within a controlled experimental environment and to compare the extent of fluorescence distribution on a macroscopic and cellular level between 5-ALA, FNa, and ICG, ultimately discerning illumination characteristics and the reliability of the agents. We utilized relevant mouse glioma models and the latest version of wide-field clinical-grade neurosurgical operating microscopes equipped with fluorescence detection systems and confocal microscopy technologies to visualize fluorescent agents simultaneously, thus making comparisons more consistent and clinically relevant.

## 2 Materials and methods

### 2.1 Study planning

Experimental procedures were performed under the guidelines and regulations of the National Institutes of Health Guide for the Care and Use of Laboratory Animals. Procedures were approved by the St. Joseph’s Hospital and Medical Center Institutional Animal Care and Use Committee. All animals were kept in the St. Joseph’s Hospital and Medical Center animal care facility. The rooms were temperature and humidity controlled and maintained under a 12-hour light-dark cycle. The animals were fed standard rodent chow and had free access to water.

### 2.2 Glioma models

#### 2.2.1 GL261 glioma model in C57BL/6-luc2 mice

The GL261 mouse glioma cells (Division of Cancer Treatment and Diagnosis of the National Cancer Institute) (19) were made bioluminescent via Lentiphos HT System with Lenti-X HT Packaging Mix (Clontech Laboratories, Inc., Mountain View, CA) and FUW-GL plasmid (Dr. J. B. Rubin, Washington University, St. Louis, MO). We injected 10–12-week-old female C57BL/6-luc2 mice with 2 μl of 1–2 × 10^7^ cells/ml 2.0 mm below the brain surface as previously described (20, 21).

#### 2.2.2 GB3 glioma model in CrTac: NCr-Foxn1^nu^ nude mice

We used 5–6-week-old CrTac: NCr-*Foxn1* ^nu^ nude mice (Taconic Biosciences, Germantown, NY) with a patient-derived cell line (GB3) that was established previously from a primary glioblastoma at the Barrow Neurological Institute and was validated as having glioma stem cell features (22). GB3 cells were made fluorescent by transduction with premade lentiviral particles (Amsbio, Cambridge, MA) expressing red fluorescence protein (RFP)-Luc and then were selected using blasticidin (2 μg/ml^−1^) as previously described (23). Orthotopic cell transplants were performed by injecting 2 μl of dissociated cells at a density of 100,000 cells/μl in the right striatum, as described previously (24).

#### 2.2.3 IUE model in ICR CD-1 mice

Gliomas were induced in ICR CD-1 mice using a clustered regularly interspaced short palindromic repeats (CRISPR)–mediated gene-editing method by *in utero* electroporation (IUE) with guide RNA pX33 constructs that target and delete genes encoding neurofibromin 1, tumor protein p53, and phosphatase and tensin homolog. Preparation and validation of the model were previously described in detail (25). Two groups of animals with IUE tumor models were made: one group with and one group without the use of CAG-mRFP vector (Addgene, Watertown, MA) to express RFP for visualization of tumor cell fluorescence (the IUE +RFP group and the IUE -RFP group, respectively) (26).

### 2.3 Animal surgery

The animals were anesthetized with a xylazine (80 mg/kg) and ketamine (10 mg/kg) cocktail. 5-ALA (Sigma-Aldrich, Inc., St. Louis, MO) was injected intraperitoneally 2–3 hours before surgery (5 mg in 200 μl of 1 × phosphate-buffered saline) according to previously established protocol (27). Intravenous injection of FNa (20 mg/kg) and ICG (20 mg/kg) was performed simultaneously, immediately at the start of the procedure. Then, craniotomy was performed, and the brain tumor was exposed for intraoperative surface imaging. Animals were euthanized, with subsequent brain removal and immediate coronal slicing for further imaging. The average duration of FNa and ICG circulation was approximately 30 minutes.

Days of mouse surgery were determined based on previously established active tumor growth phase for each tumor model or if neurological symptoms or significant weight loss occurred. GL261-bearing mice were operated on 20–30 days after implantation, IUE mice were operated on postpartum days 56–112, and GB3 mice were operated on 137–139 days after implantation.

### 2.4 Fluorescence imaging with operating microscope

Fresh mouse brains were sliced immediately after animal sacrifice in the coronal plane (Adult Mouse Brain Slicer Matrix, Zivic Instruments, Pittsburgh, PA), placed on a flat surface, and investigated under the operating microscope (Kinevo, Carl Zeiss AG, Oberkochen, Germany). We used white light imaging mode for the gross image. Fluorescence modules BLUE 400, YELLOW 560, and INFRARED 800 were used for visualization of PpIX, FNa, and ICG, respectively. The microscope objective was set at a 200-mm focal distance to increase the gain of fluoresce detection. Images were recorded with the internal microscope camera at manually set exposure times of 15, 30, or 60 frames per second to avoid oversaturation or undersaturation. This setup was used to replicate the intraoperative scenario.

### 2.5 Rapid *ex vivo* confocal laser scanning microscopy

Coronal cuts of mouse brains were placed on 35-mm glass-bottom petri dishes (MatTek Corp., Ashland, MA) and scanned on the LSM 710 DUO CLSM (Carl Zeiss AG, Oberkochen, Germany) equipped with EC Plan-Neofluar 10×/0.3 and Plan-ApoChromat 20×/0.75 microscope objectives (Carl Zeiss Microscopy, LLC, White Plains, NY). A separate channel was used for each fluorophore imaging to avoid bleed-through. All samples were imaged for PpIX, FNa, and RFP (for RFP-expressing tumors). In addition, some samples were imaged for 488 reflectance for surface imaging of myelin fibers. Some samples were treated with 1 drop of Hoechst live cell nuclear stain (Hoechst 33342, Thermo Fisher Scientific, Waltham, MA), incubated for 20 minutes, washed gently, and imaged immediately.

For PpIX imaging, we used 405-nm laser and 609–667-nm detection window with 405-nm beamsplitter. For spectroscopic imaging, the sample was interrogated with 405-nm laser with 423–726-nm detection window and 405-nm beamsplitter using lambda mode and measurements taken at the desired regions of interest. For Hoechst imaging, we used 405-nm excitation laser with 412–500-nm detection window and 405-nm beamsplitter. For reflection mode, we used 488-nm laser with 482–497-nm detection window with MBS T80/R20 beamsplitter. For RFP imaging in IUE mice, we used 594-nm excitation with 602–710-nm detection window and 488/594-nm beamsplitter. For RFP imaging in GB3 mice, we used 560-nm excitation laser with 575–710-nm detection window and 488/561-nm beamsplitter. For FNa imaging, we used 488-nm excitation laser and 493–546-nm detection window with 488-nm beamsplitter. First tile scans of the whole brain slice were obtained with 5-μm axial resolution, and then smaller regions were scanned at higher magnification. Tile scans, Z-stacks, and individual images were analyzed in Fiji software (28).

### 2.6 Histology

Coronal brain slices were oriented in cassettes, fixed in 8% paraformaldehyde, paraffin-embedded, sliced, and stained with hematoxylin and eosin. Digital tiled scans were created by scanning slides with an Aperio VERSA microscope system (Leica Biosystems, Deer Park, IL); scans were then analyzed using Aperio ImageScope software (Leica Biosystems, Deer Park, IL).

### 2.7 Statistical analysis

#### 2.7.1 Analysis of the wide-field fluorescence images

Digital images were analyzed independently by at least 2 research fellows experienced in imaging analysis. For semiquantitative comparison, the frequencies for areas of fluorescence were assessed as follows. True-positive (TP) fluorescence score was assigned if there was a relevant area of fluorescence in a tumor area that was confirmed by RFP signal or corresponding histology findings. False-negative (FN) fluorescence score was assigned for an image with absent fluorescence in a tumor area that was confirmed by RFP signal or corresponding histology findings. False-positive (FP) fluorescence score was assigned if there was detectable significant fluorescence in normal brain areas without RFP signal or in normal brain based on histological data. Each brain slice image was graded either 1 (presence of event) or 0 (absence of event).

During analysis of operating microscope data, GL261 tumors were visible on white light mode images as well as with PpIX and FNa modes. All slices with visible tumor were amenable for grading and analysis. In GB3 tumors, no PpIX signal was detected on a first assessment, so only images from 8 samples that were later confirmed on RFP imaging to have tumors were used for analysis. In IUE +RFP tumors, we observed bright red fluorescence in YELLOW 560 mode, which corresponded to the reddish staining of the brains on gross images and is likely related to RFP expression. This bright red fluorescence did not allow assessment of FNa fluorescence in the center of the tumors; therefore, we did not assess TP FNa rate in this model. However, we were able to evaluate for FN FNa fluorescence in normal brain areas devoid of bleed-through RFP signal and also for FN FNa fluorescence in areas where RFP signal was dim and did not oversaturate the image.

PpIX fluorescence intensity was measured in areas of identified tumors in GL261, IUE +RFP, and IUE -RFP tumors. FNa fluorescence intensity was measured in GL261 and IUE -RFP tumors. ICG fluorescence intensity was measured in GL261 tumors. The contralateral nontumor hemisphere served as a control showing background signal intensity for calculation of the tumor-to-background (TBR) fluorescence intensity ratios.

For GL261 tumors, we measured the surface area of tumor visible on the gross image as well as areas of PpIX, FNa, and ICG fluorescence. For IUE +RFP tumors, we also analyzed the area of RFP signal and area of PpIX fluorescence. We did not assess FNa fluorescence area in the IUE +RFP model because of an inability to reliably detect positive FNa signal within the tumor because of RFP bleed-through.

#### 2.7.2 Semiquantitative analysis of the confocal fluorescence images

Analysis of percentages of TP, FN, and FP areas on the confocal fluorescence images was conducted similarly as described above for GL261, GB3, IUE +RFP, and IUE-RFP gliomas.

#### 2.7.3 Data presentation

Data were presented as mean and standard deviation. The 2-tail Wilcoxon signed rank test was used for pairwise comparison of dependent variables, Mann-Whitney test for independent variables, and chi-square test for the binominal data. Differences were considered significant when p<0.05. Image analysis was performed using Fiji software, and statistical analysis was performed in GraphPad Prism version 8.2.0 for Mac (GraphPad Software, San Diego, CA).

## 3 Results

### 3.1 Wide-field fluorescence imaging

#### 3.1.1 GL261 gliomas

**Figure 1:**
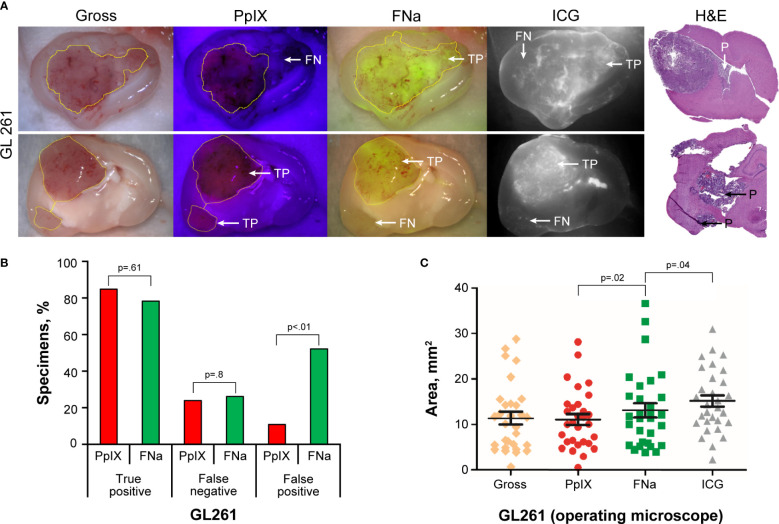
GL261 gliomas visualized using operating microscope with various fluorescent filters. **(A)** Examples of coronal slices of mouse brains interrogated under white light view (gross) as well as protoporphyrin IX (PpIX), fluorescein sodium (FNa), and indocyanine green (ICG) fluorescence. In most cases, PpIX, FNa, and ICG highlighted the bulk of the tumors well. However, there was a tumor area that was highlighted by FNa fluorescence but not by PpIX (*top row*) and vice versa (*bottom row*). Samples stained with hematoxylin and eosin (H&E) were used as a reference to identify location of the tumors, indicated as positive (P). **(B)** Comparison of the percentages of true-positive (TP), false-negative (FN), and false-positive (FP) areas between PpIX and FNa fluorescence. The y-axis indicates the percentage of specimens in which TP, FN, and FP fluorescence were identified. Among 46 GL261 brain samples (18 animals), 39 (85%) had TP findings, 11 (24%) had FN findings, and 5 (11%) had FP findings of areas of tumor with PpIX fluorescence. With FNa fluorescence, 36 (78%) samples had TP findings of areas of tumor, 12 (26%) had FN findings, and 24 (52%) had FP findings. PpIX fluorescence showed significantly fewer FP fluorescent areas (p<0.01). **(C)** Quantitative comparison of tumor areas (n=30 samples) as identified from gross white light picture (mean [SD] area, 11.4 [7.5] mm^2^), PpIX fluorescence (11.1 [6.5] mm^2^), FNa fluorescence (13.1 [8.4] mm^2^), and ICG fluorescence (15.4 [6.9] mm^2^). A larger area was highlighted with FNa than with PpIX (p=0.02, by Wilcoxon matched-pairs signed rank test), and a smaller area was highlighted with FNa than with ICG (p=0.04, by Wilcoxon matched-pairs signed rank test). *Used with permission from Barrow Neurological Institute, Phoenix, Arizona*.

GL261 gliomas showed strong fluorescence for all 3 fluorophores, and the tumor was visible on gross imaging (**Figure 1A**). However, there were areas within the tumor where PpIX was very dim or not visible that were FNa positive and vice versa. Semiquantitative analysis showed that FNa and PpIX were equally effective in highlighting the bulk of the tumor, because the TP rate did not differ significantly (p=0.61) and some tumor areas were missed with similar frequency (p=0.8). However, compared to PpIX, FNa produced significantly more FP staining of normal areas surrounding the tumor, areas of surgical trauma, or areas of reduced BBB (p<0.01) (**Figure 1B**). GL261 was the only tumor with detectable and reliably quantifiable ICG signal. ICG resulted in a significantly larger fluorescence area than FNa, and both were larger than the PpIX positive area (**Figure 1C**).

#### 3.1.2 GB3 gliomas

**Figure 2:**
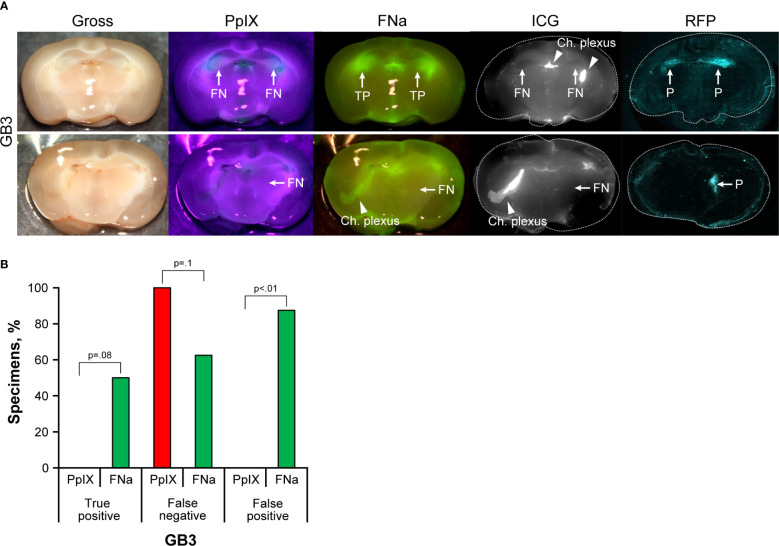
GB3 gliomas visualized using operating microscope with various fluorescent filters. **(A)** Examples of coronal slices of mouse brains visualized using operating microscope with various fluorescent filters for gross view and protoporphyrin IX (PpIX), fluorescein sodium (FNa), and indocyanine green (ICG) fluorescence. No visible PpIX fluorescence was detected using the operating microscope. Some tumors showed visible FNa fluorescence (*top row*); however, most were FNa negative (*bottom row*). ICG did not show visible fluorescence in GB3 tumors but highlighted the choroid plexus. FNa stained normal tissue at the base of the brain, at the choroid plexus (Ch. Plexus), and around the third ventricle. Red fluorescence protein (RFP) visualized under a confocal microscope was used as a reference to identify tumors, indicated as positive (P). **(B)** Comparison of the percentages of true-positive (TP), false-negative (FN), and false-positive (FP) areas between PpIX and FNa fluorescence. Among 8 GB3 samples analyzed (6 animals), 0 (0%) had TP findings, 8 (100%) had FN findings, and 0 (0%) had FP findings of areas of tumor with PpIX fluorescence. With FNa fluorescence, 4 (50%) samples had TP findings, 5 (63%) had FN findings, and 7 (88%) had FP finding of areas of tumor. More TP fluorescent areas were seen with FNa fluorescence than with PpIX (p<0.01); however, these areas were seen in only 50% of the tumors (p=0.08). *Used with permission from Barrow Neurological Institute, Phoenix, Arizona*.

GB3 gliomas were small infiltrative tumors and were not discernable on gross images under the operating microscope. Therefore, RFP fluorescence was used to identify samples with tumor and to delineate tumor location (**Figure 2A**). Among 18 animals (141 slices), in 8 slices of 6 animals, tumors were identified on histology or RFP analysis via confocal imaging and used for subsequent analysis. The remainder of brain slices did not show tumor tissue due to the small tumor size. PpIX was not detected using wide-field operating microscopy. FNa was detected in 4 (50%) of the 8 studied samples. The paraventricular areas that corresponded to the choroidal plexus were stained with FNa as well, accounting for a high FP percentage of 88% (7 of 8 slices). ICG was identified in the choroid plexus but was not detected in any of the tumors (**Figure 2B**).

#### 3.1.3 IUE gliomas

**Figure 3:**
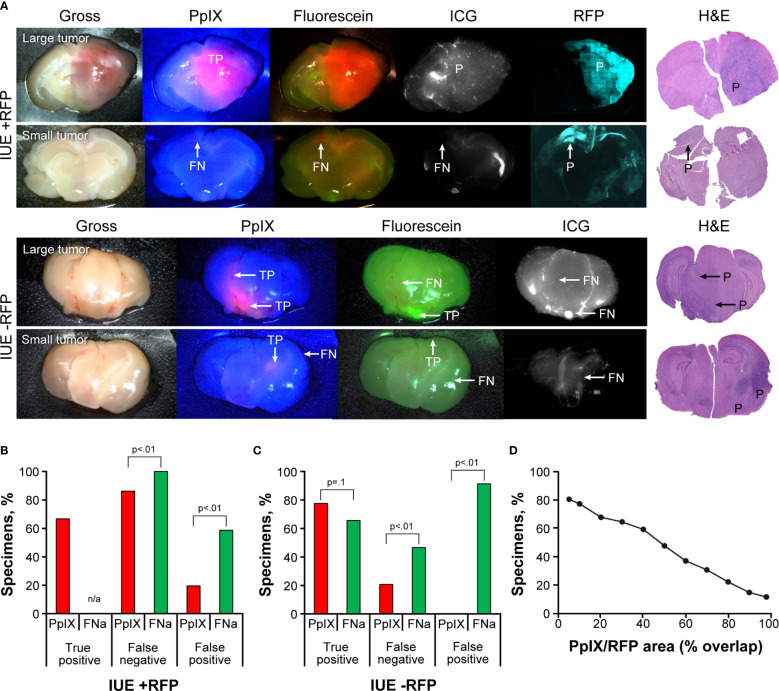
*In utero* electroporation glioma model with and without red fluorescence protein (IUE +RFP and IUE -RFP, respectively) visualized using operating microscope with various fluorescent filters. **(A)** Examples of gross view, protoporphyrin IX (PpIX), fluorescein sodium (FNa), and indocyanine green (ICG) fluorescence. In IUE +RFP tumors, PpIX highlighted the bulk of the tumors; however, the peripheries of large and small tumors were PpIX negative. True-positive (TP), false-negative (FN), and positive (P) findings are indicated where appropriate. **(B)** Comparison of percentages of TP, FN, and false-positive (FP) areas between PpIX and FNa fluorescence. We were able to calculate the percentage of visualized areas and determine the staining pattern of PpIX in the IUE +RFP model compared with the IUE -RFP model. Strong fluorescence of RFP under YELLOW 560 mode did not permit reliable detection of TP FNa in IUE +RFP tumors; therefore, it was not calculated in this model. However, in small tumors, where bright FP FNa fluorescence of the normal brain areas was obvious and RFP signal was not as bright and did not oversaturate the image, we were able to assess FP and FN FNa percentages. We used RFP fluorescence visualized under confocal microscope and hematoxylin and eosin (H&E)–stained sections as a reference to identify tumor locations. Among 87 IUE +RFP samples (13 animals), 58 (67%) had TP findings, 75 (86%) had FN findings, and 17 (20%) had FP findings of areas of tumor with PpIX fluorescence. Areas of tumor with TP FNa fluorescence were not calculated because of overlapping strong RFP fluorescence. FN areas were observed in 87 (100%) samples, and FP areas were observed in 51 (59%) samples. Significantly fewer FN and FP fluorescent areas were found with PpIX fluorescence than with FNa (both p<0.01). **(C)** Among 58 IUE -RFP samples (7 animals), 45 (78%) had TP findings, 13 (22%) had FN findings, and 0 (0%) had FP findings with PpIX fluorescence. With FNa fluorescence, 38 (66%) samples had TP findings, 27 (47%) samples had FN findings, and 53 (91%) samples had FP findings. Significantly fewer FN and FP fluorescent areas were observed with PpIX fluorescence than with FNa (both p<0.01). **(D)** Relation between percentage of specimens and overlap between PpIX and RFP fluorescence. PpIX-positive fluorescence covering 80% of the tumor was observed in only 22% of specimens, 50% overlap was observed in 47% of specimens, and at least 20% overlap was observed in 68% of specimens. *Used with permission from Barrow Neurological Institute, Phoenix, Arizona*.

IUE gliomas were diffuse gliomas that were difficult to discern on gross imaging. Only large tumors that distorted normal anatomy and IUE +RFP tumors that had slight red tinge were identifiable by the unaided eye; however, the true extension of the tumors remained undiscernible on gross images. PpIX fluorescence was bright in the center of the large tumors but was dim or not visible in small tumors. FNa was visible in IUE -RFP tumors, but the contrast to the surrounding brain was less prominent with FNa than with PpIX. ICG did not highlight IUE tumors (**Figure 3A**). Although FNa had positive staining of the tumor bulk in most of the IUE -RFP tumor areas and there was no difference in the TP rate between FNa and PpIX (66% [38/58] vs 78% [36/46], p=0.1), FNa had significantly higher FN and FP rates compared to PpIX in both IUE -RFP and IUE +RFP groups (both p<0.01, **Figures 3B, C**).

The RFP signal was easily discernable under YELLOW 560 operating microscope detection mode, making it easier to identify reference tumor margins for comparison with PpIX but preventing reliable TP FNa identification; this likely contributed to the FP rate being lower than in IUE -RFP cohort. In 41 of 87 (47%) IUE +RFP brain slices, the PpIX highlighted at least 50% of RFP-positive tumor area (**Figure 3D**).

### 3.2 Confocal fluorescence microscopy

#### 3.2.1 GL261 gliomas

**Figure 4:**
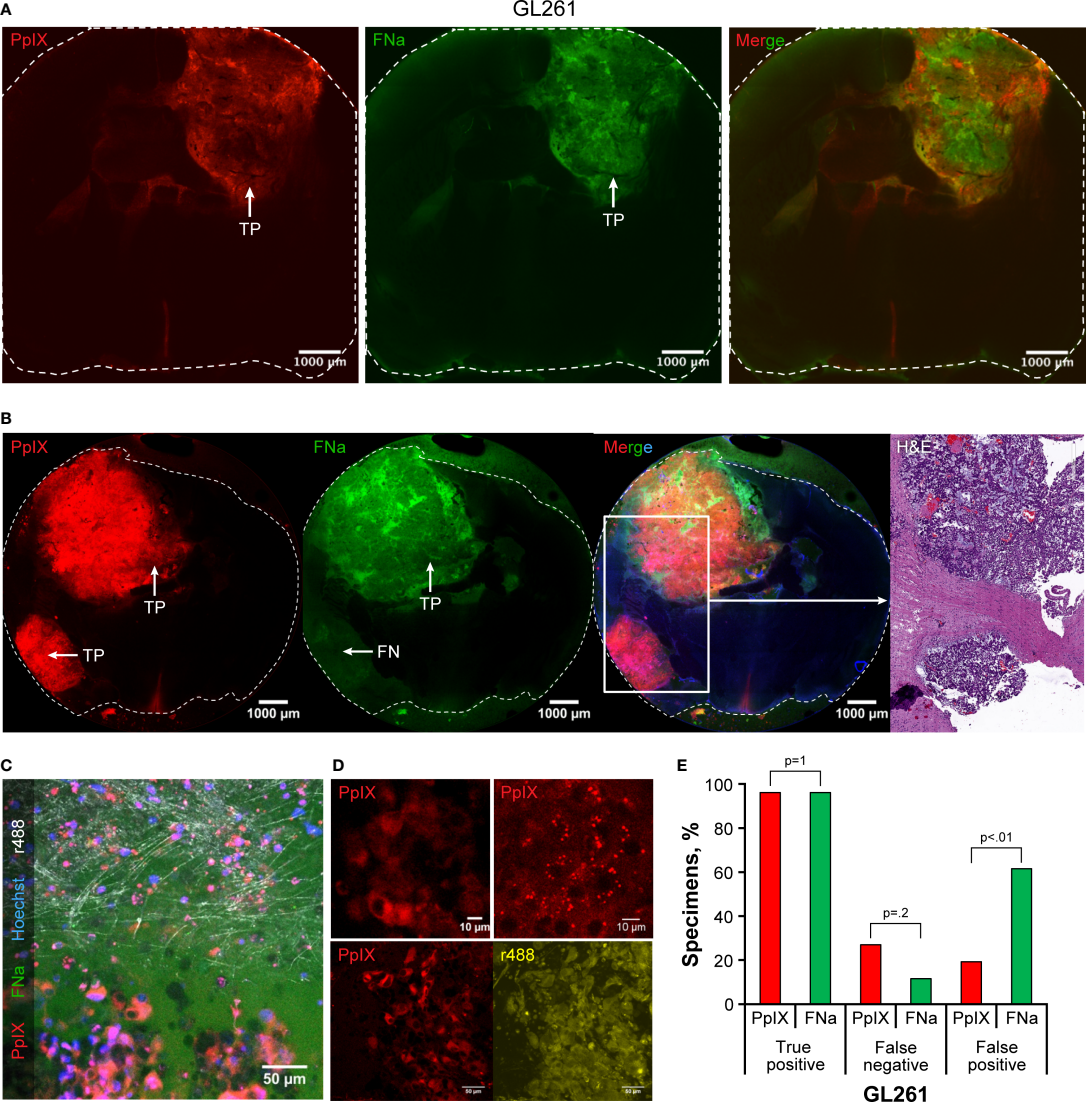
Confocal imaging of coronal brain slices with GL261 gliomas. **(A)** Most tumors presented as large uniform mass highlighted equally well by protoporphyrin IX (PpIX) and fluorescein sodium (FNa). **(B)** In some areas, however, PpIX highlighted tumor areas that were not highlighted by FNa. Presence of the tumor in false-negative (FN) FNa areas is evident in the hematoxylin and eosin (H&E) stain. **(C)** A confocal image at the tumor border shows large, atypical cells with intracellular PpIX accumulation at the bottom of the image. FNa has typical extracellular distribution, creating shadows of unstained cells. Note sparse PpIX-positive cells in the normal brain at the top of the image. Reflection (r488) shows normal brain axons in white, and Hoechst stain highlights nuclei in blue. **(D)** GL261 tumor cells have various PpIX staining patterns. Note heterogeneous staining of the cells in the top left image and intracellular granules with PpIX on the top right image. Another field of view at the bottom demonstrates that not all GL261 cells accumulate PpIX and that the degree of accumulation varies. The contours of all cells are shown in reflection r488 mode for reference. **(E)** Quantitative comparison of the percentages of true-positive (TP), FN, and false-positive (FP) areas between PpIX and FNa fluorescence seen on confocal images. Among 26 GL261 samples (6 animals), 25 (96%) had TP findings, 7 (27%) had FN findings, and 5 (19%) had FP findings with PpIX fluorescence. With FNa fluorescence, 25 (96%) samples had TP findings, 3 (12%) samples had FN findings, and 16 (62%) samples had FP findings. A lower percentage of FP areas was found with PpIX fluorescence than with FNa (p<0.02). *Used with permission from Barrow Neurological Institute, Phoenix, Arizona*.

Both FNa and PpIX exhibited bright fluorescence that highlighted most of the tumor (**Figures 4A, B**). Variable PpIX intracellular fluorescence was observed. With time, intracellular PpIX fluorescence transformed from diffuse intracellular to a granular appearance, most likely due to photobleaching and diffusion of the cytoplasmic PpIX. Granular sparse PpIX fluorescence was also detectable in the normal brain area (**Figure 4C**). The appearance of PpIX in the tumor cells was diffuse or granular intracytoplasmic with various amounts of PpIX in different cells (**Figure 4D**). The percentages of TP (96% [25/26] vs 96% [25/26], p>0.99) and FN (27% [7/26] vs 12% [3/26], p=0.2) were similar when comparing PpIX and FNa; however, PpIX produced significantly less FP than FNa (19% [5/26] vs 62% [16/26], p<0.01) (**Figure 4E**).

#### 3.2.2 GB3 gliomas

**Figure 5:**
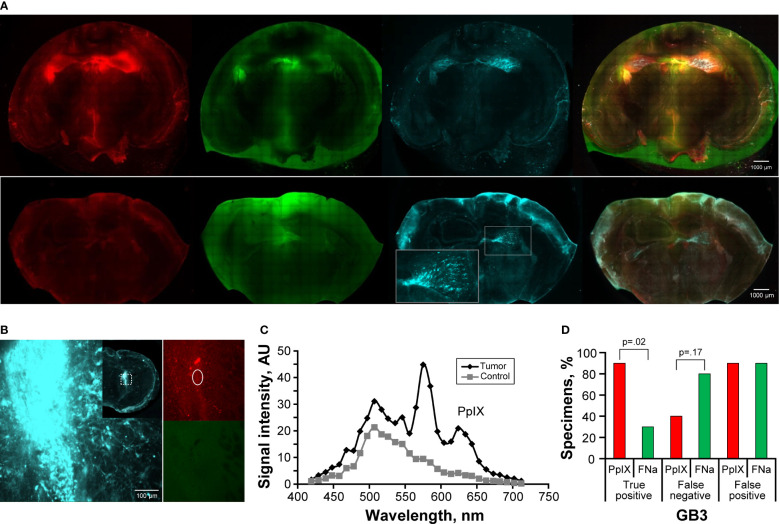
Confocal imaging of coronal brain slices with GB3 gliomas. **(A)** The top row shows tumors in both hemispheres with detectable protoporphyrin IX (PpIX) and fluorescein (FNa) fluorescent signal. Note false-positive (FP) PpIX and FNa fluorescence in the areas with permeable blood-brain barrier, such as the third ventricle, ependymal area, and choroidal plexus. Red fluorescence protein (RFP) was used as a reference for locating the tumor. The bottom row shows an example of a smaller GB3 glioma with false-negative (FN) PpIX and FNa fluorescence. The inset shows RFP-positive tumor enlarged. **(B)** Example of RFP-positive tumor in the basal ganglia with mildly positive PpIX signal and negative FNa fluorescence. The inset shows a large, tiled scan of the coronal brain slice and square area from which the image was taken. The oval area shows the region from which spectroscopic measurements shown in panel C were taken. **(C)** Diagram showing spectroscopic confirmation of PpIX presence (smaller peak at 633 nm) and negative brain area as a control in a GB3 tumor sample shown in panel **(B)**. **(D)** Quantitative comparison of the percentages of true-positive (TP), FN, and FP areas between PpIX and FNa fluorescence seen on confocal images. Among 10 GB3 samples (6 animals), 9 (90%) had TP findings, 4 (40%) had FN findings, and 9 (90%) had FP findings with PpIX fluorescence. With FNa fluorescence, 3 (30%) samples had TP findings, 8 (80%) samples had FN findings, and 9 (90%) samples had FP findings. A higher percentage of TP areas was found with PpIX fluorescence than with FNa (p=0.02). Low PpIX accumulation in the GB3 model, as evidenced by low PpIX fluorescence signal, made it difficult to identify tumor. *Used with permission from Barrow Neurological Institute, Phoenix, Arizona*.

PpIX signal was detectable in the tumor area in this tumor model. However, because of the low PpIX signal intensity within the tumor, a high gain setting led to detection of FP red fluorescence from the other brain areas (**Figure 5A**). PpIX presence in the tumors was confirmed by spectral imaging with a peak at 633 nm (**Figures 5B, C**). Fluorescence within the tumor region was more frequently detectable with PpIX imaging than with FNa imaging (TP 90% [9/10] vs 30% [3/10], p=0.02). FNa fluorescence was associated with a higher FN rate than PpIX (80% [8/10] vs 40% [4/10], p=0.17); however, because of the high gain setting necessary to detect both fluorophores, the FP rate for both was high (90% [9/10] vs 90% [9/10], p>0.99) (**Figure 5D**).

#### 3.2.3 IUE gliomas

**Figure 6:**
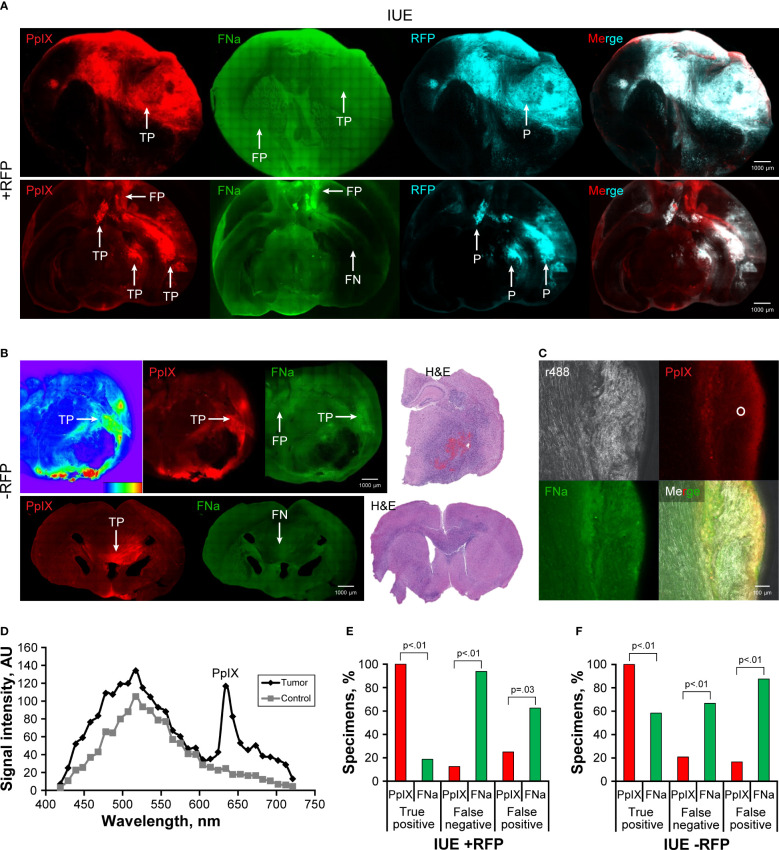
Confocal imaging of coronal brain slices with *in utero* electroporation gliomas with and without red fluorescence protein (IUE +RFP and IUE -RFP, respectively). **(A)** Top row demonstrates example of a large IUE +RFP glioma with positive protoporphyrin IX (PpIX) fluorescence findings. Fluorescein sodium (FNa) was detected both in the tumor and in normal brain, which made it impossible to differentiate the tumor border. RFP was used as a reference for identification of the tumor. The bottom row shows large, complex IUE +RFP glioma highlighted with PpIX fluorescence. Some of the tumor accumulated FNa, but most of the tumor did not show significant FNa accumulation compared to the normal brain. Also note the false-positive (FP) PpIX and FNa fluorescence of the base of the brain and at the third ventricle. **(B)** The top row shows an example of a large IUE -RFP glioma that had significant PpIX accumulation of various degrees. Heatmap diagram demonstrates intensity of PpIX signal (red indicates high intensity, and blue indicates low intensity). Note the large necrotic core devoid of PpIX and FNa fluorescence as well as areas with FP FNa fluorescence. FNa signal was increased in some tumor areas but did not provide sufficient contrast to identify the tumor border. The bottom row shows smaller IUE -RFP glioma in the corpus callosum with true-positive (TP) PpIX and false-negative (FN) FNa fluorescence. Hematoxylin and eosin (H&E)–stained slices were used as a reference to identify the location of IUE -RFP gliomas. **(C)** A higher magnification confocal image shows accumulation of PpIX and FNa in the glioma on the right side of the specimen, with a reflectance image showing organized normal brain white matter nerve fibers on the left. Circle shows the area where spectroscopic measurements were taken. **(D)** Diagram shows an example of spectroscopic confirmation of PpIX presence in an IUE -RFP sample. **(E)** Quantitative comparison of the TP, FN, and FP percentages between PpIX and FNa seen on confocal images. Among 16 IUE +RFP samples (8 animals), 16 (100%) had TP findings, 2 (13%) had FN findings, and 4 (25%) had FP findings with PpIX fluorescence. With FNa fluorescence, 3 (19%) samples had TP findings, 15 (94%) samples had FN findings, and 10 (63%) samples had FP findings. PpIX fluorescence was associated with a higher percentage of TP areas (p<0.01) and a lower percentage of FN and FP fluorescent areas (p<0.01 and p=0.03, respectively). **(F)** Among 24 IUE -RFP samples (15 animals), 24 (100%) had TP findings, 5 (21%) had FN findings, and 4 (17%) had FP findings with PpIX fluorescence. With FNa fluorescence, 14 (58%) samples had TP findings, 16 (67%) samples had FN findings, and 21 (88%) samples had FP findings. PpIX fluorescence was associated with a higher percentage of TP areas and a lower percentage of FN and FP areas (all p<0.01). *Used with permission from Barrow Neurological Institute, Phoenix, Arizona*.

Both IUE +RFP and IUE -RFP tumors were diffuse, invasive, and demonstrated high PpIX fluorescence intensity compared to GB3, resulting in less FP PpIX fluorescence (**Figure 6A**). PpIX fluorescence was not uniform and varied significantly, with high signal at the invasive tumor core and lower signal at the necrotic part and in the peripheral infiltration zone, which correlated with the histological data (**Figure 6B**). PpIX fluorescence was confirmed by spectral imaging with a peak at 633 nm (**Figures 6C, D**). In both IUE +RFP and IUE -RFP tumors, PpIX was a more reliable indicator of tumor cell presence than FNa based on superior TP, FP, and FN rates (**Figures 6E, F**).

### 3.3 TBR in wide-field fluorescent imaging

**Figure 7:**
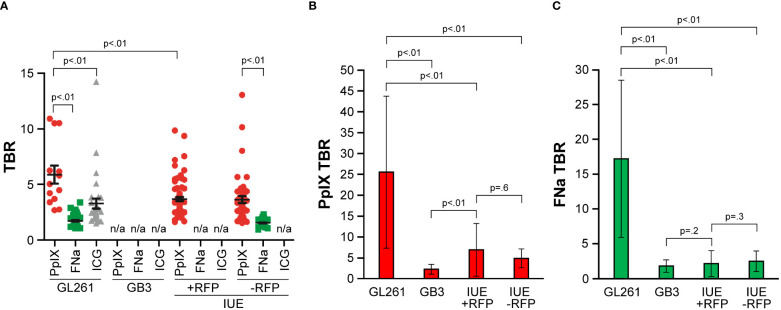
Comparison of fluorescence intensities as measured by tumor-to-background ratio (TBR) among GL261, GB3, and *in utero* electroporation with and without red fluorescence protein (IUE +RFP and IUE -RFP, respectively) gliomas. **(A)** Operating microscopy showed significant differences in TBR among different fluorophores (p<0.01). Among GL261 tumors, the protoporphyrin IX (PpIX) TBR (mean [SD], 5.6 [3.0]; n=13) was significantly higher than the TBR for fluorescein sodium (FNa) (mean [SD], 1.4 [0.5]; n=63) or indocyanine green (ICG) (mean [SD], 2.9 [2.5]; n=31) (both p<0.01). GB3 gliomas have no significant PpIX fluorescence and were excluded from analysis. Mean (SD) PpIX fluorescence TBR in IUE +RFP gliomas was 3.4 (1.6) (n=75). In IUE -RFP gliomas, mean (SD) TBR was higher for PpIX fluorescence (3.4 [2.1]; n=46) than for FNa (1.3 [0.3]; p<0.01). GL261 gliomas showed higher TBR than both IUE models (both p<0.01). Differences in FNa TBR between GL261 and IUE -RFP gliomas were not significant (p=0.17). **(B)** Comparison of TBR of PpIX fluorescence detected by confocal microscope. **(C)** Comparison of TBR of FNa fluorescence detected by confocal microscope. *Used with permission from Barrow Neurological Institute, Phoenix, Arizona*.

The TBR of PpIX was similar in IUE +RFP and IUE -RFP gliomas and was slightly less than the TBR of PpIX in GL261 gliomas (**Figure 7A**). In the GL261 model, the TBR with PpIX was higher than with FNa and ICG. TBR calculation was not performed in GB3 tumors for all 3 fluorophores, in IUE +RFP for FNa, and in both IUE +RFP and IUE -RFP for ICG because of uncertain fluorescent staining patterns that prevented reliable tumor identification.

### 3.4 TBR in laser scanning confocal fluorescent imaging

Under confocal visualization, the PpIX fluorescence TBR (mean [SD], 25.5 [18.2]) was approximately 5 times brighter in the GL261 model than in the IUE model (p<0.01) (**Figure 7B**). There were no significant differences in PpIX fluorescence TBR between the IUE +RFP (mean [SD], 6.9 [6.3]) and IUE -RFP (mean [SD], 4.8 [2.6]) models, and in both of these models it was higher than in the GB3 model (2.2 [1.1]) (p<0.01).

Under confocal visualization, FNa fluorescence TBR (mean [SD], 17.2 [11.3]) was approximately 7 times brighter in the GL261 model than in the IUE model (p<0.01) (**Figure 7C**). There were no significant differences in mean (SD) FNa fluorescence TBR between the IUE +RFP (2.2 [1.9]) and IUE -RFP (2.5 [1.5]) models (p=0.3), and there were no significant differences in FNa fluorescence TBR between either of these models and the GB3 model (1.8 [0.9]) (p=0.2 and p=0.6, respectively).

The TBR was significantly higher with PpIX than with FNa in the IUE +RFP (p=0.02) and IUE –RFP (p=0.04) models. In the GL261 tumors, the TBRs with PpIX and FNa were equally high (p=0.09), and in the GB3 model, they were equally low (p=0.2).

## 4 Discussion

### 4.1 Interpretation of study results within the context of the current literature

In an effort to elucidate which of the commonly used fluorophores is more effective in optimizing visualization of glioma margins, we performed simultaneous fluorescent labeling using FNa, 5-ALA, and ICG. Because the clinical intraoperative situation may not provide sufficient time, conditions, or opportunity for complete study, we sought to assess the activity of neurosurgical fluorophores within a controlled experimental environment as applied to relevant glioma models. The ability of fluorophores to highlight tumor tissue was assessed based on their macroscopic fluorescence and microscopic distribution visualized by a state-of-the-art neurosurgical operating microscope and a confocal microscope in 3 experimental glioma models with various grades of malignancy. Simultaneous labeling of brain tumors has been reported previously, but to the best of our knowledge this is the first systematic *in vivo* investigation using both wide-field operating microscopy and confocal systems.

We used 3 murine models of gliomas. The GL261 tumor model is considered the gold-standard syngeneic model, has an immune component for experimental work mimicking high-grade gliomas, and has been successfully used to visualize fluorophores in previous studies (16, 29–32). The GB3 tumor model is a patient-derived glioma in immunocompromised mice that has preserved stem cell properties of the human glioma cells, as opposed to the syngeneic mouse glioma, but lacks immune features (22). The IUE model is a secondary glioblastoma model that leads to development of a brain glioma with progressively increased grade (25). When it is smaller and early in development, the IUE model histologically resembles a grade 2 and grade 3 glioma, and as it grows larger over time, it develops grade 4 features, such as necrosis. The rationale for the IUE model is to study the diagnostic accuracy of FNa and PpIX in a diffuse syngeneic tumor model that is not created surgically, without previous surgical trauma to the brain. IUE and GB3 tumor models are invasive, show lower grade in early stages, and have a highly diffuse growth pattern compared with that of the GL261 model, which exhibits more focal growth. Our observations suggested that GL261 could be viewed as a fast and reproducible model for initial gross assessment of fluorophore guidance analogous to the glioblastoma bulk tissue. The IUE tumor model, although less consistent with respect to the location of the developed tumor, is far more comprehensive, displaying a variety of tissue conditions, including various degrees of tumor infiltration, BBB disruption, hypervascularization, and necrosis.

We demonstrate that, in the highly proliferative and less diffuse GL261 tumor model that resembles the core of glioblastoma, PpIX and FNa fluorescence are associated with similarly high TP rates, and both can miss some of the tumor (presence of FN areas), but FNa is associated with a higher FP rate for staining surrounding edematous tissue, choroid plexus, and dura. These findings corroborate with the high positive predictive value of wide-field operating microscope fluorescence detection of FNa [96% (33)] and PpIX [97.5% (34)] for detection of glioblastoma tissue in humans. It should be noted that FN areas of the tumor occurred in both PpIX and FNa staining and not always at the same regions. PpIX and FNa fluorescence, therefore, were complimentary to each other in identification of the tumor margins in this model under both wide-field microscopy and confocal imaging.

In contrast to GL261, GB3 tumors were small infiltrative tumors analogous to the infiltrative margin of gliomas. PpIX fluorescence was detected by confocal microscopy but not with the wide-field operating microscope. It was previously suggested that PpIX accumulation below the detection threshold of the operating microscope could be detected even in low-grade gliomas with quantitative wide-field fluorescence imaging (35), spectroscopy (36), fluorescence life-time imaging (37), or other probe-based or near-view systems (27, 38). Similarly, the highly invasive IUE glioma model demonstrated higher TP and lower TN rates with PpIX than with FNa under confocal imaging. In GB3 and IUE models, decreasing PpIX and FNa fluorescence over the tumor margins was apparent, especially under the operating microscope. Meanwhile, confocal high-resolution imaging allowed for more reliable detection of PpIX fluorescence at the margins of GB3 and IUE gliomas. Confocal imaging, therefore, may be helpful in detection of PpIX in low-grade tumors along with other adjuncts (39). FNa fluorescence was not as specific as PpIX in determining the exact location of the tumor and was patchy, highlighting some nonspecific brain areas, cerebrospinal fluid, and paraventricular areas along with dura and choroid plexus. In most specimens, FP FNa fluorescence intensity of nontumorous regions was brighter than TP fluorescence in the tumor regions, making it hard to reliably distinguish tumor location. Identification of cellular morphology was possible using FNa as a background contrast in our confocal experiments. Given that intraoperative confocal endomicroscopy at a similar magnification level to that used in our study with FNa is being used for the assessment of tumor margins in clinical settings (27, 40–42), it is important to assess optimal imaging parameters for visualization of tissue microstructure because doing so facilitates the differentiation of tumor and nontumor areas (43) and may augment clinical decision making. We have previously extensively studied confocal imaging of the GL261 model with confocal endomicroscopy (43), and our current data show a similar histological pattern (**Figure 4**). However, we were not able to reliably evaluate the microstructural histological picture with FNa as a contrast in GB3 or IUE models. Based on our experience, acute FNa administration results in slightly better contrast between the bright extracellular space and dark cells (44), but more data are necessary.

### 4.2 Existing literature on combined use of 5-ALA and FNa

There have been several reports on simultaneous use of 5-ALA and FNa in small patient samples. Schwake et al. investigated combined use of 5-ALA and FNa in 4 patients using a Leica M530 OH6 microscope with FL400 and FL560 filters (Leica Biosystems, Deer Park, IL) (45). They compared early (35 minutes prior to durotomy) and acute FNa administration. With early administration, FNa highlighted glioblastoma tissue but was also visible in dura, cerebrospinal fluid, and normal brain. In low-grade glioma, 5-ALA–induced PpIX fluorescence was not visible, whereas FNa showed patchy fluorescence in the tumor and also stained surrounding normal brain. In 2 other glioblastoma cases, PpIX fluorescence was visible in the invading margin, but FNa fluorescence was FN (45). These findings were confirmed by Yano et al., who assessed histological samples of gliomas after dual labeling with 5-ALA and high-dose (20 mg/kg) FNa. Interestingly, they used a D-light system (Karl Storz 20133620, Karl Storz, Tuttlingen, Germany) for excitation of PpIX, whereas both PpIX red fluorescence and yellow FNa staining were observed without special filters (14). Yano et al. demonstrated that FNa was more prevalent in the tumor bulk, whereas PpIX was more prevalent in the tumor margins, where there were fewer vessels but still adequate tumor cells. They determined that PpIX had a higher sensitivity and negative predictive value for detecting tumor boundaries than FNa, but tumor cells still existed beyond the areas visualized by both agents, indicating that neither method was perfect in measuring the tumor boundary (14). In our study of the 3 tumor models, we found that FNa did not localize in tumor cells distant to the tumor core, whereas PpIX successfully highlighted them. Although invading tumor cells were missed by both techniques on gross imaging, confocal microscopy findings indicated that a higher percentage of invading tumor cells were successfully highlighted with PpIX than with FNa.

Suero Molina et al. investigated simultaneous dual labeling with the goal of improving tumor visualization by PpIX fluorescence by brightening the background tissue with a low dose of FNa (46). YELLOW 560 filter was used for visualization, combined with various light intensities from the microscope that excited FNa in the tissues and a separate fiber optic blue light source (D-light C System) for excitation of PpIX. Again, FNa was not helpful for detecting additional tumor but rather provided enhanced depiction of background against which PpIX fluorescence was perceived (46). The same group described a novel filter set, YB 475, that was compatible with the Zeiss Pentero microscope and allowed simultaneous excitation of PpIX and FNa with xenon light via a bandpass 390–475-nm window and simultaneous detection of fluorescence from both fluorophores (47). Improved background visualization from this technique led to better appreciation of blood vessels and easier bleeding control without the need to frequently switch to white light mode (47).

Suero Molina et al. (48) then moved away from using FNa as a background enhancer and presented a new BLUE 400 AR filter that allowed for more ambient background light to be seen and resulted in better background visualization while maintaining high specificity of PpIX detection compared to the BLUE 400 filter (Carl Zeiss Meditec, Oberkochen, Germany). Comparable multispectral imaging technology (ARveo, Leica Microsystems Inc, Deerfield, IL) that visualized PpIX fluorescence on a bright background has been described (49).

Della Puppa et al. (50) described observations of PpIX and FNa fluorescence in 3 patients using the latest Zeiss Kinevo microscope, similar to one used in our study. The Kinevo operating microscope employed in our study has an improved built-in camera and optical filters that allowed photographic documentation and more reliable comparison of wide-field fluorescence images than results seen with the operating microscope in previous reports. Our observations in the GL261 glioma model provide insights of PpIX and FNa fluorescence at the cellular level to demonstrate that they are complimentary to each other at the tumor margins of newly diagnosed glioblastoma as observed by Della Puppa et al. (50) and Suero Molina et al. (46) Based on our findings, the poor specificity of FNa in GB3 and IUE tumor tissues creates a potential for misidentifying healthy tissue as cancerous. According to recent research, it appears as though FNa is more suitable for assessment of BBB integrity and tumor-associated disruption of the BBB, which may be a feature of many pathological intracranial processes, including, most importantly, malignant brain tumors (51). 5-ALA–induced PpIX is more often detected intracellularly, and to a lesser extent in extracellular fluorescence of metabolically active pathological tissue, as well as in some normal brain areas. This study, along with previous reports, demonstrates that there are multiple variables that affect detection of PpIX and FNa fluorescence intensities, including biological and optical variables (52–55) that result in the absence of fluorescence in some tumor areas as well as FP staining of the normal brain tissue with both fluorophores.

### 4.3 ICG for identification of tumor border

ICG has been studied for tumor margin visualization in multiple cancer types. Studies have shown that ICG is effectively taken up in adrenal cancers (56), various head and neck cancers (57), and the sentinel lymph nodes of patients with breast or endometrial cancer (58, 59). In the scheme of neurological tumors, Hansen et al. were able to demonstrate that ICG accurately depicted rat glioma models within 1 mm with gross tissue staining (15). Cho et al. described a SWIG technique for brain tumor visualization using a higher dose of ICG administered 24 hours before imaging and a highly sensitive near-infrared surgical detection system, VisionSense Iridium exoscope (VisionSense, Philadelphia, PA) (60). Although our study demonstrated bright ICG fluorescence in the GL261 glioma model, similar to previous reports with SWIG (61), we were not successful in visualizing ICG in GB3 or IUE models; therefore, we did not analyze ICG signal in those groups. Also, ICG signal was barely visible 2–3 hours after injection in GL261 tumors. SWIG technique could potentially improve visualization of ICG in more invasive tumor models and is a subject for further studies. We did not evaluate microscopic details of ICG accumulation because the confocal microscope used was not equipped with a near-infrared excitation laser. Intracellular ICG localization has been previously demonstrated (62), and Martirosyan et al. (16) and Zehri et al. (17) demonstrated intracellular accumulation of ICG in a GL261 glioma model showing definitive tumor borders and associated microvascular and subcellular structures using a prototype dedicated confocal laser endomicroscope. This growing compilation of successful usage of ICG suggests the potential of ICG to outperform a surgeon’s subjective identification of tumor margins *in vivo*. Although the literature shows that ICG has the features of strong cellular uptake and sensitive tumor margin identification, we observed only first-pass ICG accumulation in our murine glioma models, most of which corresponded to the vascular phase of ICG and did not highlight diffuse GB3 and IUE tumors.

### 4.4 Dosages of fluorescent drugs and timing of imaging

We used dosages of FNa and 5-ALA that are comparable to those previously reported in animal studies with 5-ALA (27, 44, 63–65) and FNa (32, 43, 44, 66, 67). These are higher than human doses due to differences in metabolism. The FNa dose (20 mg/kg) was used based on our experience with confocal and wide-field imaging, in which it was found that this dose produces better, more reliable staining than a lower 5-mg/kg dose in an animal model (43, 44, 68). Additionally, because GL2 and IUE tumors are lower grade, we were expecting higher FNa doses to produce better images than lower doses. With respect to ICG, the maximal human dose is approximately 10 mg/kg, and the usual clinical dose uses a 25-mg vial per injection, which results in a dose of approximately 0.3 mg/kg (15). Early experiments with ICG in animal glioma found that a dose of 60–120 mg/kg is necessary for visible staining with ICG (fluorescence was not used) within 1 hour after injection (15, 69). Earlier, our laboratory had used lower doses of 0.4 mg/kg, which were sufficient for confocal endomicroscopy (16, 70), but in the current study, we found that a 0.4-mg/kg dose resulted in little if any tumor fluorescence under the operating microscope in infrared 800 mode. Other studies have shown that higher doses of ICG (5 mg/kg and 2.5 mg/kg) showed maximum fluorescence intensity and TBR at 1 hour after intravenous injection with an *in vivo* fluorescence imaging system through the intact skull, which correlates with our data (61, 71). Lee’s group demonstrated that absolute ICG intensity in the tumor is less at 24 hours than at 1 hour; however, overall target/background ratio is somewhat more specific, likely because ICG clears from normal tissues. Therefore, Lee’s group have chosen this 24-hour imaging time point and named it “second-window ICG imaging” (61, 71). Our experience with various ICG dosages and timing of imaging during the first hour after injection showed that supramaximal doses produced better fluorescence intensity with the operating microscope. Therefore, we have chosen a dose of 20 mg/kg administered at the start of the procedure, approximately 30 minutes prior to animal sacrifice. Lower doses in our pilot experiments did not produce reliable GL261 glioma fluorescence. Although images with higher ICG doses would potentially be brighter, we believe such doses are too toxic for any meaningful clinical use.

### 4.5 Limitations of the study

This is an animal study, and scalability to the human brain should therefore be considered when evaluating the findings. However, our primary goal was to look for microscopic differences at the border region, for which murine glioma models provide valuable insight. We also assumed that simultaneous (parallel) administration of 5-ALA and FNa did not affect the findings for either of the fluorophores. Further clinical assessment of these fluorophores is warranted to confirm the findings of our study, with some preliminary clinical data recently published (50).

Spectra of FNa, PpIX, and RFP may have some crosstalk. To minimize this limitation, we performed separate experiments with IUE without RFP and utilized spectral imaging using a confocal microscope to confirm spectral signature of fluorophores. Unlike in the IUE model, the RFP signal in the GB3 model did not have such a visible fluorescence under the YELLOW 560 mode, nor was there significant crosstalk with FNa and PpIX during confocal microscope imaging, which may be explained by differences in RFP used in these different models.

FNa is known to produce false-positive staining because of surgical trauma, especially if that trauma occurs soon after injection, while FNa is still in the circulation (72, 73). Although false-positive findings could not be completely excluded, we have specifically tried to minimize FNa extravasation caused by surgical trauma, and after trying various surgical techniques, we have chosen to slice the brain immediately after careful removal from the cranium. The absence of FNa fluorescence in the normal brain areas in a well-defined GL261 tumor model confirms the lack of significant trauma-related extravasation, whereas areas that are known to have some FNa (e.g., choroid plexus, ependyma, and dura) are clearly distinguishable. Moreover, functional-based tumor resection that allows for preservation of eloquent brain function is imperative for tumor surgery (74) and should be combined with fluorescence guidance to prevent unexpected residual and to guide decisions in functionally eloquent areas.

We used a state-of-the-art neurosurgical microscope set at the most clinically relevant and optically favorable position for fluorescence detection. However, sensitivity to PpIX and ICG fluorescence might be less than in advanced research imaging systems used by others, potentially accounting for less efficacy of ICG detection in our study (61).

Analysis of FP, FN, and TP areas was conducted by research fellows competent in fluorescence imaging, and it aimed at identification of large meaningful tumor areas and fluorescence staining that would be considered significant for fluorescence-guided surgery. However, findings were nonetheless based on subjective assessment.

## 5 Conclusion

With acute ICG administration at the induction of anesthesia, fluorescence was visible in GL261 gliomas only within the first few minutes after injection and was not detected in GB3 or IUE tumors using wide-field operating microscope imaging. 5-ALA, ICG, and FNa demonstrated highly accurate comparable detection of neoplastic tissue with wide-field imaging in a high-grade murine glioma (GL261), although FNa and ICG had more FP staining areas. Diagnostic accuracy of PpIX and FNa in the GB3 and IUE models was inferior compared to GL261 tumors. In invasive intermediate-grade tumors (IUE tumor model), confocal imaging was more sensitive for detection of low PpIX and FNa signal than wide-field operating microscope imaging, resulting in better diagnostic accuracy rates and higher TBR. Moreover, confocal imaging allowed for detection of PpIX signals in a lower-grade GB3 glioma that were not detectable with wide-field operating microscope imaging.

These data have practical clinical implications for the treatment of what, it must be remembered, is a brain-systemic disease. If aiming for a maximal total resection, simultaneous administration of 5-ALA and FNa may provide complimentary information for optimal tumor margin detection, especially when utilizing wide-field operating microscope imaging for a high-grade tumor. Although 5-ALA and FNa highlighted the gross tumor bulk, neither 5-ALA nor FNa worked perfectly in delineating all tumor tissue or individual cells at the margins for wide-field or cellular-level microscope imaging, thus highlighting the need for improvements in current methods and novel solutions for fluorescence guidance in surgery of malignant gliomas.

## Data availability statement

The raw data supporting the conclusions of this article will be made available by the authors, without undue reservation.

## Ethics statement

The animal study was reviewed and approved by St. Joseph’s Hospital and Medical Center Institutional Animal Care and Use Committee.

## Author contributions

Conceptualization: EB, MP. Supervision and advising: VB, JL, MP. Design: EB, LB, IA, MP. Literature search: EB, LB, EL-M. Experimental studies: EB, LB, IA, DH, SM, MP. Data analysis: EB, LB, IA, TK, KY, BD. Statistical analysis: TK, KY, DH. Manuscript preparation: EB, IA, MP. Manuscript editing and review: EB, IA, MP. All authors contributed to the article and approved the submitted version.
